# Optimizing Diagnosis and Surgery for Pure Anterior Mediastinal Cysts: Insights from a Single-Centre Study

**DOI:** 10.1093/icvts/ivaf288

**Published:** 2025-12-12

**Authors:** Arnaud Rieg, Axel Rouch, Pierre Rabinel, Claire Renaud, Noémie Vantaux, Mathilde Cazaux, Laurent Brouchet, Romain Vergé

**Affiliations:** Department of Thoracic Surgery, Toulouse University Hospital, Toulouse 31400, France; Department of Thoracic Surgery, Toulouse University Hospital, Toulouse 31400, France; Department of Thoracic Surgery, Toulouse University Hospital, Toulouse 31400, France; Department of Thoracic Surgery, Toulouse University Hospital, Toulouse 31400, France; Department of Thoracic Surgery, Toulouse University Hospital, Toulouse 31400, France; Department of Thoracic Surgery, Toulouse University Hospital, Toulouse 31400, France; Department of Thoracic Surgery, Toulouse University Hospital, Toulouse 31400, France; Department of Thoracic Surgery, Toulouse University Hospital, Toulouse 31400, France

**Keywords:** pure anterior mediastinal cysts, thymic cysts, thymoma

## Abstract

**Objectives:**

Pure anterior mediastinal cysts (pAMC) are uncommon and typically benign lesions. In contrast to solid anterior mediastinal tumours, there are no standardized diagnostic or management guidelines, despite a small but clinically significant risk of malignancy. The goal of this study was to identify preoperative factors associated with malignancy in order to develop a clinical algorithm to guide the diagnosis and management of pAMC.

**Methods:**

This retrospective single-centre study was conducted at Toulouse University Hospital and included patients diagnosed with pAMC between January 2012 and June 2025. Logistic regression was used to identify preoperative predictors of thymoma or germ cell tumours.

**Results:**

A total of 70 patients were diagnosed with pAMC, 26 of whom underwent surgical resection. On multivariate analysis, hypermetabolism on positron emission tomography–computed tomography (PET-CT) was significantly associated with a final pathological diagnosis of thymoma or germ cell tumour (odds ratio, 43.21 [2.81-663.93], *P* = .007). Lesion size greater than 4 cm was also associated with malignancy on univariate analysis (odds ratio: 10.50 [1.50-73.67], *P* = .02), though this did not remain significant in multivariate analysis. Based on these findings, we proposed a decision-making algorithm incorporating PET-CT as a first-line investigation.

**Conclusions:**

Hypermetabolism on PET-CT is a strong preoperative predictor of malignancy in lesions presenting as pAMC. Prospective multicentre studies are warranted to validate additional prognostic markers and optimize imaging strategies for the management of pAMC.

## Graphical abstract

**Figure:**
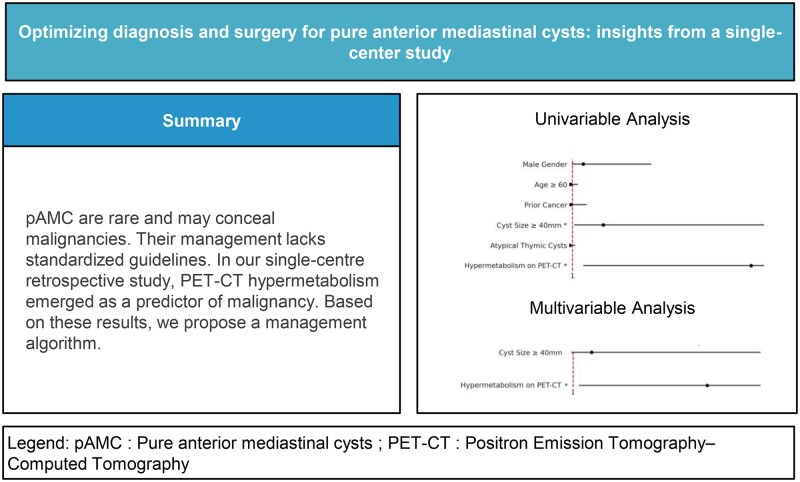

## INTRODUCTION

Pure anterior mediastinal cysts (pAMC) are uncommon lesions that may be either congenital—arising during embryogenesis—or acquired later in life.^1^ They are most often identified incidentally on images obtained for unrelated indications, because the majority remain asymptomatic.^2^ However, when sufficiently large, pAMC may cause compressive symptoms due to the effect of the mass on adjacent mediastinal structures.^3^

In contrast to the well-established diagnostic and therapeutic protocols for anterior mediastinal solid tumours,^4–7^ there are currently no standardized guidelines for the evaluation or management of pAMC. This lack of consensus is clinically relevant, because histological analysis typically confirms their benign nature, yet a small but notable proportion may harbour malignancy—most commonly thymomas, and more rarely germ cell tumours.^8^

Although several case series have described the radiologic and pathological features of pAMC,^9–11^ standardized algorithms for diagnostic assessment and therapeutic decision-making remain lacking. Consequently, the timing and necessity of surgical intervention for these lesions are still debated and highly variable across centres.

The primary objective of this study was to identify preoperative factors predictive of malignancy or with premalignant potential, with the goal of optimizing clinical decision-making. Secondary objectives consisted of characterizing our institutional cohort and proposing a structured algorithm to guide the diagnostic and therapeutic management of pAMC.

## MATERIAL AND METHODS

### Study population

This retrospective, single-centre study was conducted at Toulouse University Hospital and included all patients diagnosed with or treated for pAMC who were discussed at the regional multidisciplinary tumour board (MTB) meetings between January 2012 and June 2025.

Patients were eligible for inclusion if they presented with radiologic findings suggestive of pAMC on computed tomography (CT). Exclusion criteria included patients with clearly solid anterior mediastinal masses or either part-solid mediastinal lesions or a definitive diagnosis of pleuropericardial or bronchogenic cysts, because these entities follow established diagnostic and therapeutic protocols that differ significantly from pAMC management.

### Diagnostic and therapeutic workflow

In our region, all patients with suspected pAMC were referred to the regional MTB, which serves as the centralized referral platform for mediastinal pathologies.

At initial presentation, patients typically underwent contrast-enhanced chest CT. The diagnostic work-up was primarily guided by the radiologic interpretation presented at the MTB, led by an expert thoracic radiologist. When findings suggested a pure cystic lesion, further imaging with magnetic resonance imaging (MRI) was routinely performed to better characterize the lesion.

Contrast-enhanced MRI was used to characterize the pAMC described on chest CT scans. The lesions typically appear hypointense on T1-weighted images and hyperintense on T2-weighted images, consistent with their fluid content. The radiologist classified pAMC as either typical or atypical cysts according to the British Thoracic Oncology Group.^7^ Cysts were deemed atypical if they exhibited enhancement, serial enlargement, progressive mural thickening, mural nodularity, mural calcification, or septation; all others were classified as typical.

A fluorodeoxyglucose positron emission tomography-computed tomography (PET-CT) scan could also be conducted to evaluate metabolic activity within the cyst wall.

Finally, in rare and ambiguous cases, image-guided percutaneous biopsy might be considered.

Following completion of the diagnostic imaging, each case was re-reviewed during the MTB meeting. In the absence of standardized guidelines for pAMC, the decision to proceed with an operation was based on expert consensus, incorporating imaging features from CT, MRI, and any hypermetabolic uptake seen on PET-CT.

In our institution, an operation was considered in cases of symptomatic cysts, several defining features of atypical cysts on MRI, PET-CT hypermetabolism, or based on patient preference. In contrast, conservative management was chosen when lesions were deemed benign, with annual MRI for the first 2 years, followed by a further MRI in the fourth year, after which the need for continued surveillance is discussed on a case-by-case basis. Surgical intervention could be reconsidered in cases where the lesion showed progression or morphological changes during follow-up.

### Operation

All patients undergoing an operation received general anaesthesia, with robotic-assisted thoracoscopic surgery (RATS) proposed as the first-line approach. The procedure was typically performed via the left side, unless the cyst was predominantly right-sided. Patients were positioned supine, with arms slightly abducted to facilitate thoracic access. The operation was carried out using the da Vinci Si or Xi robotic system (Intuitive Surgical, Sunnyvale, CA, USA), employing 3 robotic trocars and 1 assistant port, which could be enlarged if necessary to extract the surgical specimen.

In cases involving large cysts, anatomical constraints, or intraoperative concerns such as potential vascular injury, a median sternotomy was performed.

Given the potential for malignancy, a complete thymectomy was systematically carried out in all surgical cases.

### Data collection

Clinicopathologic variables—including age, gender, presenting symptoms, comorbidities, radiologic findings, and if applicable, surgical approach, operative time, postoperative complications, length of hospital stay, and final pathological diagnosis—were collected.

### Statistical methods

Categorical variables were presented as proportions and compared using Pearson’s χ^2^ test or Fisher’s exact test when sample size was less than 5. Continuous variables were expressed as mean ± SD, with the *t*-test used to compare their distributions.

Logistic regression was performed to try to identify prognostic factors for thymoma or germinal tumours. All the variables with a *P*-value less than .1 from the univariable method were analysed in a multivariable analysis.

All data analyses were conducted with 2-sided tests: a *P* value of less than .05 was considered statistically significant. Statistical analyses were performed using Stata/MP 14.1 (StataCorp LP, College Station, TX, USA).

*P* values were not adjusted for multiplicity.

The study protocol received approval from the Ethics Committee of Toulouse University Hospital (RnIPH-2025–121) and was performed in accordance the WMA Declaration of Taipei. Due to the retrospective study design, written informed consent was waived.

There were no missing data.

Complete data analysis was carried out in this study.

## RESULTS

### Preoperative characteristics

Between 2012 and 2025, a total of 70 patients with pAMC were reviewed by our regional MTB (**Table 1**). Of these, 54.3% were female, and the mean age was 60.1 years. A history of autoimmune disease was documented in 15.7% of cases; however, only 1 patient had confirmed myasthenia gravis.

**Table 1.** Characteristics of Patients, Cysts and Multidisciplinary Tumour Board for Pure Anterior Mediastinal Cysts

	Study population	Typical thymic cysts	Atypical thymic cysts	*P*-value
	n = 70 (%)	n = 26 (%)	n = 35 (%)	
Patient characteristics				
Gender (%)				
Male	32 (45.7)	14 (53.8)	12 (34.3)	0.13
Female	38 (54.3)	12 (46.2)	23 (65.7)	
Age (years)	60.1 ± 16.2	66.0 ± 11.0	58.2 ± 16.6	**0.04**
Comorbidities (%)				
Auto-immune disease	11 (15.7)	5 (19.2)	5 (14.3)	0.61
Prior cancer	17 (24.3)	6 (23.1)	9 (25.7)	0.81
Cardiovascular comorbidity	17 (24.3)	7 (26.9)	9 (25.7)	0.92
Respiratory comorbidity	16 (22.9)	6 (23.1)	9 (25.7)	0.81
Absence of symptoms (%)	66 (94.3)	25 (96.2)	32 (8.6)	0.63
Cyst characteristics				
Cyst size at first imaging (mm)	39.0 ± 26.0	36.1 ± 20.2	34.7 ± 27.0	0.83
MRI performed (%)	61 (87.1)	By definition	By definition	
Described as typical thymic cysts	26 (37.1)	–	–	
Described as atypical thymic cysts	35 (50)	–	–	
PET-CT performed (%)	60 (85.7)	20 (76.9)	32 (91.4)	0.15
With hypermetabolism on PET-CT	9 (12.9)	0 (0)	5 (14.3)	0.07
Multidisciplinary Tumour Board				
MTB proposal after imaging (%)				
Follow-up	37 (52.9)	26 (100)	8 (22.9)	**<0.001**
Operation	33 (47.1)	0 (0)	27 (77.1)	
Time between first imaging and MTB proposal (days)	316 ± 423	384 ± 458	264 ± 262	0.20
Operation performed (%)	26 (37.1)	0 (0)	20 (57.1)	**<0.001**

Abbreviations: MRI: magnetic resonance imaging; MTB: multidisciplinary tumour board; PET-CT: positron emission tomography-computed tomography.

The mean cyst diameter on initial imaging was 39 mm. MRI was performed in 87.1% of patients, whereas PET-CT was performed in 85.7%. Based on MRI results, 37.1% of the cysts were classified as typical, and 50% as atypical. Among those who underwent PET-CT imaging, hypermetabolism was observed in 12.9% of cases, with a mean standard uptake value maximum of 3.1.

Baseline clinical characteristics were comparable between patients with typical and atypical thymic cysts, with the exception of age: Patients with atypical cysts were generally younger than those with typical cysts (*P* = .04).

### Surgical and pathological characteristics

Surgical resection was recommended in 33 patients (47.1%), based on atypical radiologic features, increased metabolic activity on PET-CT scans, or other clinical concerns. Among these, 7 patients declined an operation after multidisciplinary evaluation. Notably, none of the lesions classified as typical thymic cysts exhibited progression during follow-up (mean duration, 26.1 months), and none required surgical intervention. These observations further support the indolent biological nature of these lesions.

A total of 26 patients (37.1%) ultimately underwent thymectomy to rule out a neoplastic lesion in the form of a pAMC (**Table 2**).

**Table 2.** Characteristics of Patients Who Underwent Surgical Resection for a Pure Anterior Mediastinal Cyst

	Study population
	*n* = 26 (%)
Surgical characteristics	
Motivation for surgery (%)	
Hypermetabolism on PET-CT	7 (26.9)
Several defining features of atypical thymic cyst	17 (65.4)
Others	2 (7.7)
Time between first imaging-surgery (days)	326 ± 62
Surgical approach (%)	
RATS	20 (76.9)
Open (sternotomy)	6 (23.1)
Operating time (min)	79.6 ± 4.4
Complications (%)	0 (0)
Hospital length of stays (days)	4.0 ± 0.2
Final pathological diagnosis	
Size on the surgical specimen (mm)	51.1 ± 7.3
Histological diagnosis (%)	
Benign cysts	18 (69.2)
Thymic cyst	14 (53.8)
Other cysts	4 (15.4)
Thymoma or germ cell tumours	8 (30.8)
Thymoma	4 (15.4)
Germinal tumours	4 (15.4)
Including teratoma	3 (11.5)
Including non-seminomatous germ cells tumours	1 (3.9)

Abbreviations: PET-CT: positron emission tomography-computed tomography; RATS: robotic-assisted thoracoscopic surgery.

The mean interval between initial imaging and surgical resection was 326 days. Most procedures (76.9%) were performed using a RATS approach, and 23.1% required a planned sternotomy due to lesion size or proximity to vital structures. No intraoperative conversions or vascular injuries were reported. The mean operative time was 79.6 min, and the average length of hospital stay was 4 days. No major perioperative complications occurred, with variations in length of stay primarily related to postoperative pain management.

Histopathological analysis confirmed benign cystic lesions in 18 patients (69.2%), including 14 thymic cysts, 3 bronchogenic cysts, and 1 pleuropericardial cyst. The remaining 8 patients (30.8%) were found to have neoplastic or potentially malignant lesions: 4 thymomas and 4 germ cell tumours (3 mature teratomas and 1 non-seminomatous germ cell tumour). All thymomas were early-stage (Masaoka-Koga stage I or II), with no lymph node involvement or distant metastasis.

### Predictive factors of neoplastic lesion

To optimize the selection of pAMC requiring resection, we investigated preoperative predictors of malignancy, specifically the presence of thymoma or germ cell tumours (**Table 3**).

**Table 3.** Predictive Factors of Malignant Lesions or Lesions with the Potential for Malignant Transformation (Thymoma and Germ Cell Tumours) in Pure Anterior Mediastinal Cysts

Associated factors	Univariable analysis		Multivariable analysis	
	OR [95 CI]	*P*-value	OR [95% CI]	*P*-value
Male gender	4.33 [0.74-25.29]	.10		
Age ≥ 60	0.48 [0.09-2.65]	.40		
Prior cancer	0.50 [0.05-5.36]	.57		
Cyst size ≥ 40 mm*	10.50 [1.50-73.67]	.02*	6.84 [0.47-99.72]	.16
Atypical thymic cysts	0.22 [0.03-1.74]	.15		
Hypermetabolism on PET-CT *	**56.0 [4.33-724.05]**	.002*	**43.21 [2.81-663.93]**	**.007**

Abbreviations: CI: confidence interval; OR: odds ratio; PET-CT: positron emission tomography-computed tomography.

On univariable analysis, cyst diameter equal to or greater than 40 mm on initial imaging (*P* = .02) and hypermetabolism uptake on the PET-CT (*P* = .002) scans were significantly associated with a final diagnosis of these tumours.

On multivariable analysis, despite the limited sample size, only PET-CT hypermetabolism remained an independent predictor of neoplastic pathology (*P* = .007). These findings support the integration of PET-CT imaging as a key diagnostic tool in the preoperative assessment of AMC, aiding in risk stratification and surgical decision-making (**Figure S1**).

**Figure 1.**
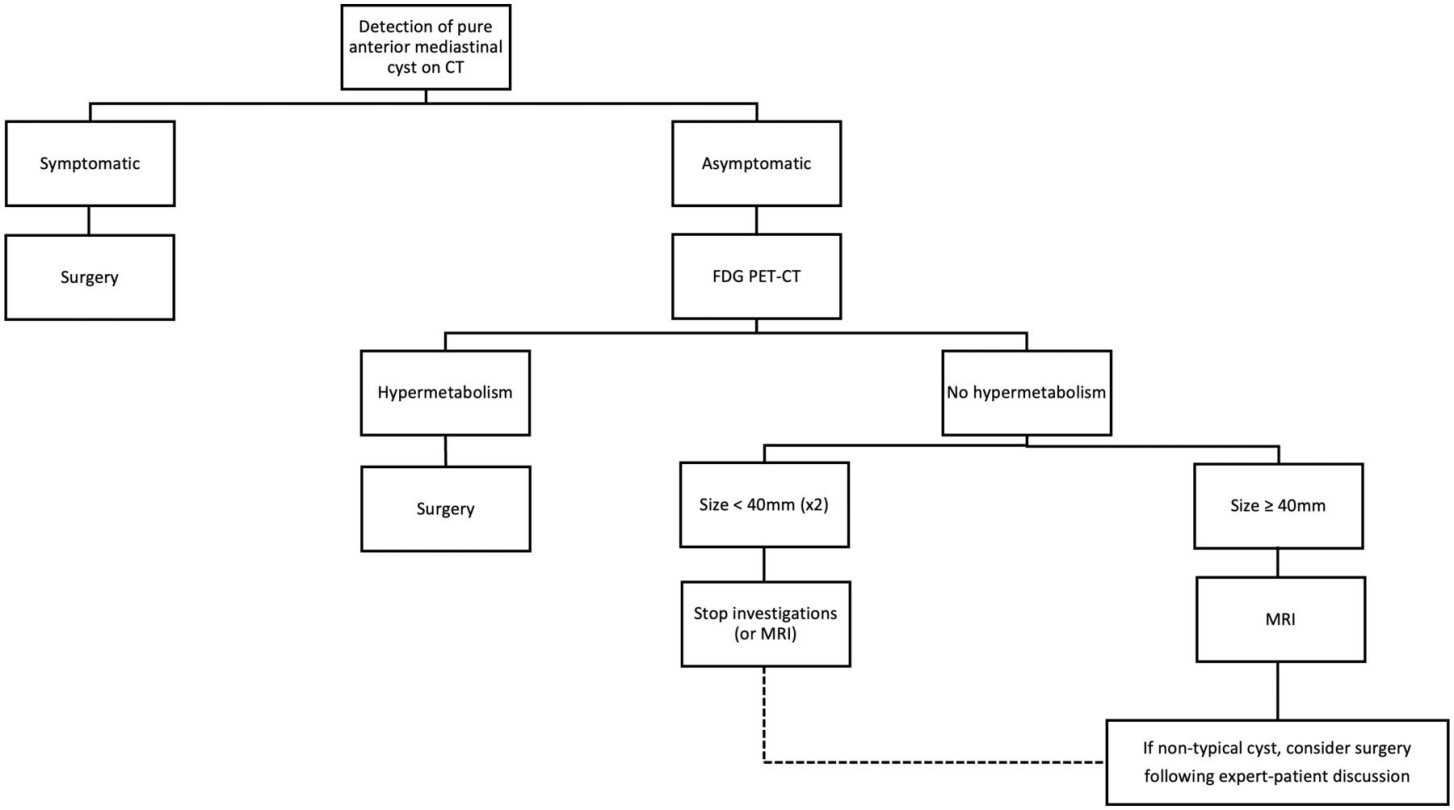
Decision-Making Algorithm for Management of and Surgery for Pure Anterior Mediastinal Cysts

### Decision-making algorithm for management of and surgery for pAMC

Based on our findings, we propose a decision-making algorithm to guide the diagnostic and therapeutic management of pAMC (**Figure 1**). Given the demonstrated predictive value of PET-CT scans, we recommend it as the first-line imaging modality when a pAMC is incidentally discovered. A positive PET-CT scan should prompt surgical resection, as it strongly suggests malignancy or malignant potential.

In cases in whom PET-CT scanning results are negative, we suggest performing an MRI to further characterize the lesion, in line with findings from the literature (**Table 4**). However, based on our data, we propose reserving MRI for cysts measuring greater than 4 cm, because this parameter was significantly associated with malignancy in univariable analysis. This stepwise approach is designed to balance diagnostic accuracy with resource utilization and patient safety.

**Table 4.** Review of the Literature on Pure Anterior Mediastinal Cysts

Year	Author	Number of pAMCs	Results
1968	Seltzer^23^	12	Only pAMC, 58% symptomatic, all multiloculated but 16% with calcifications in the wall
2001	Choi^9^	8	Unilocular cysts on CT: 50% multilocular on final pathology, only one associated with thymoma.
2003	Takeda^24^	30	About congenital cysts: 28.6% were thymic cysts (second most common congenital cyst)
2007	Tomiyama^10^	12	First-choice diagnostic accuracy for thymic cysts: 46% with CT, 71% with MRI, and 63% with CT and MRI combined
2013	McEarlean^25^	10	Benign lesions: younger patients, mostly median and shorter
2018	Yoon^1^	14	A total of 0.03% thymic cysts from 56,358 patients who underwent a low-dose chest CT scan as part of routine health check-up
2018	Proli^17^	8	PET-CT sensitivity: 83%; specificity: 58%. FDG PET-CT alone not recommended to determine malignancy
2018	Hwang^11^	3	MRI features: T2 bright and relative enhancement ratio <26.1% associated with increased rate of correct diagnosis of cyst
2019	Kim^14^	56	No significant difference of volume-doubling times between cysts and malignant tumours; non-growing lesions are not always benign
2020	Wang^22^	120	A total of 50.7% were correctly diagnosed by chest CT: 1 thymic cyst was associated with a thymoma. Surgery: low rate of complications (5.7%) and conversions (1%)
2021	Ackman^15^	140	Thymic cysts change over time in size and morphology.
2022	Choe^8^	204	A total of 19 operations for simple cysts (17 thymic cysts, 2 pericardial cysts); 8 operations for complex cysts (1 mature teratoma, 3 thymomas, 4 thymic cysts)
2022	Joubert^3^	19	Cystic lesions: 22% of all patients; 63% asymptomatic, 11% VATS resection
2024	Mayhew^19^	96	Cohort of patients studied over 10 years: none developed malignancy during follow-up
2025	Zhao^26^	70	Biphasic contrast-enhanced CT: strong diagnostic capability in differentiating thymoma from thymic cysts

Abbreviations: CT: computed tomography; FDG: fluorodeoxyglucose; MRI: magnetic resonance imaging; pAMC: pure anterior mediastinal cysts; PET-CT: positron emission tomography–computed tomography; VATS: video-assisted thoracoscopic surgery.

## DISCUSSION

### Strategy for diagnosis of pure anterior mediastinal cysts

pAMC are rare, typically benign lesions, most often discovered incidentally during imaging performed for unrelated reasons, because they are usually asymptomatic.^1^ Thus, we found only a limited number of studies focused specifically on pAMC (**Table 4**), typically as case series or as subpopulations within larger cohorts of mediastinal masses.

In this way, their management is not standardized, and patient care typically involves MRI and PET-CT imaging, with decisions regarding surveillance or operative treatment largely based on local clinical practices.^12^ Although very large pAMC can occasionally become symptomatic due to compression of adjacent structures—thereby justifying operative intervention—the optimal approach for asymptomatic cases remains controversial. This observation is particularly relevant given that some pAMC may conceal truly malignant lesions, such as thymomas or germ cell tumours, and underscores the need for a careful and individualized diagnostic strategy.^13^

Our retrospective, single-centre study presents the results of a 13-year regional cohort involving 70 pAMC and highlights the potential, central role of PET-CT in identifying lesions that are malignant or with malignant potential.

To enhance diagnostic precision, several imaging characteristics—such as cyst morphology, size, and temporal evolution—have been investigated. However, none have proven significantly reliable in distinguishing benign from malignant lesions.^14^^,^^15^

Although several studies have investigated the value of MRI in the diagnosis and follow-up of pAMC, its diagnostic accuracy remains variable. In the study by Tomiyama et al,^10^ a chest CT scan alone yielded a correct diagnosis in 46% of cases, whereas MRI increased this rate to 71%. However, in our own cohort, only 30.8% of cysts classified as atypical or suspicious on MRI were ultimately confirmed as malignant or with malignant potential on final pathology, with thymomas accounting for just 15.4% of these. Conversely, PET-CT, though not routinely employed in pAMC management,^16^ appears to be a more reliable predictor of malignancy. Proli et al.,^17^ examined 134 patients operated on for anterior mediastinal masses (cystic or solid). PET-CT positivity showed a sensitivity of 83%, specificity of 58%, positive predictive value of 90%, and negative predictive value of 94.1% for detecting malignant lesions.

Our results are consistent with those of previously published works, although higher diagnostic accuracies, up to 91%, have been reported in other studies. These discrepancies highlight the variability across cohorts and the need for multidisciplinary case-by-case assessment. Although its use in pAMC assessment is not yet standardized, integrating PET-CT findings with other imaging modalities may substantially improve the ability to detect lesions with malignant potential and guide appropriate clinical management.

Although PET-CT scans were not performed in all patients, this manuscript reflects real-world practice: Patients with typical thymic cysts often decline additional imaging, and those with atypical cysts who already had a surgical indication did not undergo PET-CT. Current guidelines recommend MRI as the first-line modality and provide limited guidance on PET-CT for cystic lesions,^16^ highlighting the relevance of our study.

In our cohort—focused exclusively on pure cystic lesions—26 patients underwent surgical resection. Notably, all lesions with hypermetabolism on PET-CT scans were confirmed to be malignant or had malignant potential. Only 1 teratoma demonstrated no FDG uptake on PET imaging, a finding that aligns with reports of other anatomical locations.^18^

Despite the limited number of operated cases, our analysis demonstrated that PET-CT positivity was a significant independent predictor of malignancy or malignant potential, with an odds ratio of 43.21 (95% CI, 2.81-663.93) in multivariable analysis.

These findings support the prioritization of PET-CT in the preoperative diagnostic workup of pAMC. In cases of negative PET-CT results, and considering data from other studies, the role of MRI may be revisited as a complementary tool, particularly when uncertainty remains.

### Surveillance and surgical considerations

The value of routine follow-up for patients with pAMC not selected for surgery remains a matter of debate. Some studies have shown that early-stage thymomas may remain radiologically stable for extended periods, suggesting that lack of progression alone cannot reliably exclude malignancy.^14^ In our cohort, none of the pAMC monitored over time demonstrated radiologic progression, consistent with results from a cohort of Mayhew et al.^19^

The choice of imaging modality for follow-up also remains unresolved. In our study, MRI was used as the primary modality for surveillance, yet its routine use is challenged by limited accessibility and, based on our findings, minimal added diagnostic value. This observation raises the question of whether its continued use in follow-up protocols is justified in the absence of specific suspicious features.

### Surgical outcomes

Surgical resection of pAMC appears to be safe and well tolerated, with a low complication rate. In comparison to existing studies on thymoma resection, minimally invasive approaches have shown non-inferiority in terms of postoperative morbidity.^20^^,^^21^ Specifically, Wang et al.^22^ reported an intraoperative complication rate of 1.1% and a postoperative complication rate of 4.9% for video-assisted thoracoscopic surgery resections of thymic cysts.

In our experience, we favoured RATS as the minimally invasive approach of choice, given its superior visualization and technical precision. Among our patients, no perioperative complications were observed, and the average length of stay was only 4 days. These results reinforce the notion that an operation, when indicated—particularly in the setting of PET-CT-avid lesions—carries minimal risk and clear potential benefit, justifying its recommendation in selected cases.

### Study limitations

This study has several limitations. It is a retrospective, single-centre analysis involving 70 patients, only 26 of whom ultimately underwent surgical resection. Consequently, some patients who were not operated on, particularly those with cysts classified as typical on MRI, may have harboured undiagnosed malignancies. Nonetheless, it is noteworthy that none of these lesions demonstrated progression during the follow-up interval, which had a mean duration of 26.1 months. The relatively small sample size also resulted in wide confidence intervals in our analysis of predictors of malignancy. Nevertheless, PET-CT hypermetabolism emerged as a statistically significant predictor in multivariable analysis, reinforcing its potential value in guiding patient management. A major limitation of our study was surgical refusal, affecting one-quarter of eligible patients. Likely reasons include the lack of clear guidelines, the low perceived malignancy risk, and concerns about the operation despite minimally invasive options and potential sternotomy. In a young, often asymptomatic population, the availability of MRI surveillance every 2 years further contributed to refusal.

To further validate our findings, a multicentre study involving a larger cohort is warranted. Such a study could help clarify inter-centre differences in management practices and facilitate the development of a more robust, standardized decision algorithm. Nevertheless, we believe that the diagnostic and therapeutic decision-making algorithm proposed in this study provides a useful starting point for improving the clinical management of pAMC.

## CONCLUSION

Our study demonstrates that PET-CT hypermetabolism is a strong predictive factor for identifying malignant lesions or lesions with malignant potential that may present as pAMC. Based on these findings, we propose a decision-making algorithm in which PET-CT may be considered as an early investigation when a pAMC is incidentally discovered on a CT scan.

Given the rarity of this pathology and the current lack of standardized guidelines, multicentre prospective studies are needed to validate these results, identify additional prognostic markers, and optimize the selection of imaging modalities. The forthcoming DETECTION project is designed within this framework.^16^ The ultimate goal is to ensure that only lesions with proven or suspected malignancy are subjected to surgical resection, thereby avoiding unnecessary procedures in patients with benign disease.

## AUTHORSHIP CONTRIBUTION STATEMENT

A.R., R.V., and L. B.: Conceptualization, Methodology, Formal analysis, Data curation, Writing—original draft, Writing—review & editing, Validation. A.R., M.C., P.R., N.V., and C.R.: Data curation, Formal analysis, Validation, Writing—review & editing. The authors report no conflicts of interest.

## SUPPLEMENTARY MATERIAL

Supplementary material is available at *ICVTS* online.

## DECLARATION OF AI-ASSISTED TECHNOLOGIES IN THE WRITING PROCESS

During the preparation of this work, the authors used ChatGPT to enhance the clarity and readability of the manuscript. After using this tool, the authors reviewed and edited the content as needed and take full responsibility for the content of the publication.

## DATA AVAILIBILITY

The data underlying this article will be shared on reasonable request to the corresponding author.

## Supplementary Material

ivaf288_Supplementary_Data

## References

[1] Yoon SH , Choi SH, Kang CH, Goo JM. Incidental anterior mediastinal nodular lesions on chest CT in asymptomatic subjects. J Thorac Oncol. 2018;13:359-366. 10.1016/j.jtho.2017.11.124 29233791

[2] Hersche CI , Lee IJ, Wu N, et al CT screening for lung cancer: prevalence and incidence of mediastinal masses. Radiology. 2006;239:586-590. 10.1148/radiol.2392050261 16641357

[3] Joubert V , Chalabreysse L, Gamondes D, Tronc F, Maury JM, Girard N. Clinical practices for non-neoplastic thymic lesions: results from a multidisciplinary cohort. Lung Cancer. 2022;166:76-83. 10.1016/j.lungcan.2022.02.004 35227953

[4] Carter BW , Benveniste MF, Madan R, et al ITMIG classification of mediastinal compartments and multidisciplinary approach to mediastinal masses. RadioGraphics. 2017;37:413-436. 10.1148/rg.2017160095 28129068

[5] Ackman JB , Chung JH, Walker CM, et al ACR appropriateness criteria imaging of mediastinal masses. J Am Coll Radiol. 2021;18:S37-S51. 10.1016/j.jacr.2021.01.007 33958117

[6] Referentiel_RYTHMIC _2022. https://www.rythmic.org/images//RYTHMIC/Doc/Referentiel_RYTHMIC_2022.pdf.

[7] Evison M , Robinson SD, Sharman A, et al; BTOG Thymic Malignancies Special Interest Group. Making an accurate diagnosis of anterior mediastinal lesions: a proposal for a new diagnostic algorithm from the BTOG Thymic Malignancies Special Interest Group. Clin Radiol. 2024;79:404-412. 10.1016/j.crad.2024.03.003 38565483

[8] Choe J , Lee SM, Ahn Y, Kim CH, Seo JB, Lee HY. Characteristics and outcomes of anterior mediastinal cystic lesions diagnosed on chest MRI: implications for management of cystic lesions. Insights Imaging. 2022;13:136. 10.1186/s13244-022-01275-8 35976511 PMC9385942

[9] Choi YW , McAdams HP, Jeon SC, et al Idiopathic multilocular thymic cyst: CT features with clinical and histopathologic correlation. AJR Am J Roentgenol. 2001;177:881-885. 10.2214/ajr.177.4.1770881 11566694

[10] Tomiyama N , Honda O, Tsubamoto M, et al Anterior mediastinal tumors: diagnostic accuracy of CT and MRI. Eur J Radiol. 2009;69:280-288. 10.1016/j.ejrad.2007.10.002 18023547

[11] Hwang EJ , Paek M, Yoon SH, et al Quantitative thoracic magnetic resonance criteria for the differentiation of cysts from solid masses in the anterior mediastinum. Korean J Radiol. 2019;20:854-861. 10.3348/kjr.2018.0699 30993936 PMC6470082

[12] Cooley-Rieders K , Van Haren RM. Mediastinal thymic cysts: a narrative review. Mediastinum. 2022;6:33-33. 10.21037/med-22-25 36582977 PMC9792833

[13] Shen X , Jin Y, Shen L, Sun Y, Chen H, Li Y. Thymoma and thymic carcinoma associated with multilocular thymic cyst: a clinicopathologic analysis of 18 cases. Diagn Pathol. 2018;13:41. 10.1186/s13000-018-0719-7 29945642 PMC6020440

[14] Kim H , Yoon SH, Kim J, et al Growth of thymic epithelial tumors and thymic cysts: differential radiological points. Thorac Cancer. 2019;10:864-871. 10.1111/1759-7714.13016 30793538 PMC6449257

[15] Ackman JB , Chintanapakdee W, Mendoza DP, Price MC, Lanuti M, Shepard JAO. Longitudinal CT and MRI characteristics of unilocular thymic cysts. Radiology. 2021;301:443-454. 10.1148/radiol.2021203593 34427460

[16] Marcuse F , Dumoulin D, Hartemink K, et al The DETECTION project part 1: an international Delphi survey about diagnostics and treatment of anterior mediastinal cystic lesions. Lung Cancer. 2025;199:108055. 10.1016/j.lungcan.2024.108055 39657307

[17] Proli C , De Sousa P, Jordan S, UK Thoracic Surgery Research Collaborative, et al A diagnostic cohort study on the accuracy of 18-fluorodeoxyglucose (^18^ FDG) positron emission tomography (PET)-CT for evaluation of malignancy in anterior mediastinal lesions: the DECiMaL study. BMJ Open. 2018;8:e019471. 10.1136/bmjopen-2017-019471 PMC5829887 29437755

[18] Feldman DR. State-of-the-art management of germ cell tumors. Am Soc Clin Oncol Educ Book. 2018;38:319-323. 10.1200/EDBK_201139 30231334 PMC6607431

[19] Mayhew J , Kong M, Bahl A, Kohat D, Wing L, Benamore R. Behaviour of simple thymic cysts over time; is surveillance required? Clin Radiol. 2024;79:956-961. 10.1016/j.crad.2024.08.025 39294035

[20] Jurado J , Javidfar J, Newmark A, et al Minimally invasive thymectomy and open thymectomy: outcome analysis of 263 patients. Ann Thorac Surg. 2012;94:974-982. 10.1016/j.athoracsur.2012.04.097 22748641

[21] Luzzi L , Corzani R, Ghisalberti M, et al Robotic surgery vs. open surgery for thymectomy, a retrospective case-match study. J Robot Surg. 2021;15:375-379. 10.1007/s11701-020-01109-z 32632562

[22] Wang X , Li Y, Chen K, Yang F, Wang J. Clinical characteristics and management of primary mediastinal cysts: a single‐center experience. Thorac Cancer. 2020;11:2449-2456. 10.1111/1759-7714.13555 32677753 PMC7471030

[23] Seltzer RA , Mills DS, Baddock SS, Felson B. Mediastinal thymic cyst. Dis Chest. 1968;53:186-196. 10.1378/chest.53.2.186 5638292

[24] Takeda S , Miyoshi S, Akashi A, et al Clinical spectrum of primary mediastinal tumors: a comparison of adult and pediatric populations at a single japanese institution. J Surg Oncol. 2003;83:24-30. 10.1002/jso.10231 12722093

[25] McErlean A , Huang J, Zabor EC, Moskowitz CS, Ginsberg MS. Distinguishing benign thymic lesions from early-stage thymic malignancies on computed tomography. J Thorac Oncol. 2013;8:967-973. 10.1097/JTO.0b013e3182904bc2 23608816 PMC3978781

[26] Zhao L , Li J, Liu Y, Zhao W, Cai H, Cao L. CECT-based simplified scoring model for differentiating low-risk thymomas from hyper-attenuating thymic cysts: a STARD compliant article. Medicine (Baltimore). 2025;104:e44453. 10.1097/MD.0000000000044453 40922239 PMC12419334

