# Supplementary material for: Optimizing Diagnosis and Surgery for Pure Anterior Mediastinal Cysts: Insights from a Single-Centre Study
Source: Interdiscip Cardiovasc Thorac Surg. 2025 Dec 12;41(1):ivaf288. doi: 10.1093/icvts/ivaf288 (PMC12774464; doi:10.1093/icvts/ivaf288)
Supplement: ivaf288_Supplementary_Data [file ivaf288_supplementary_data.docx]

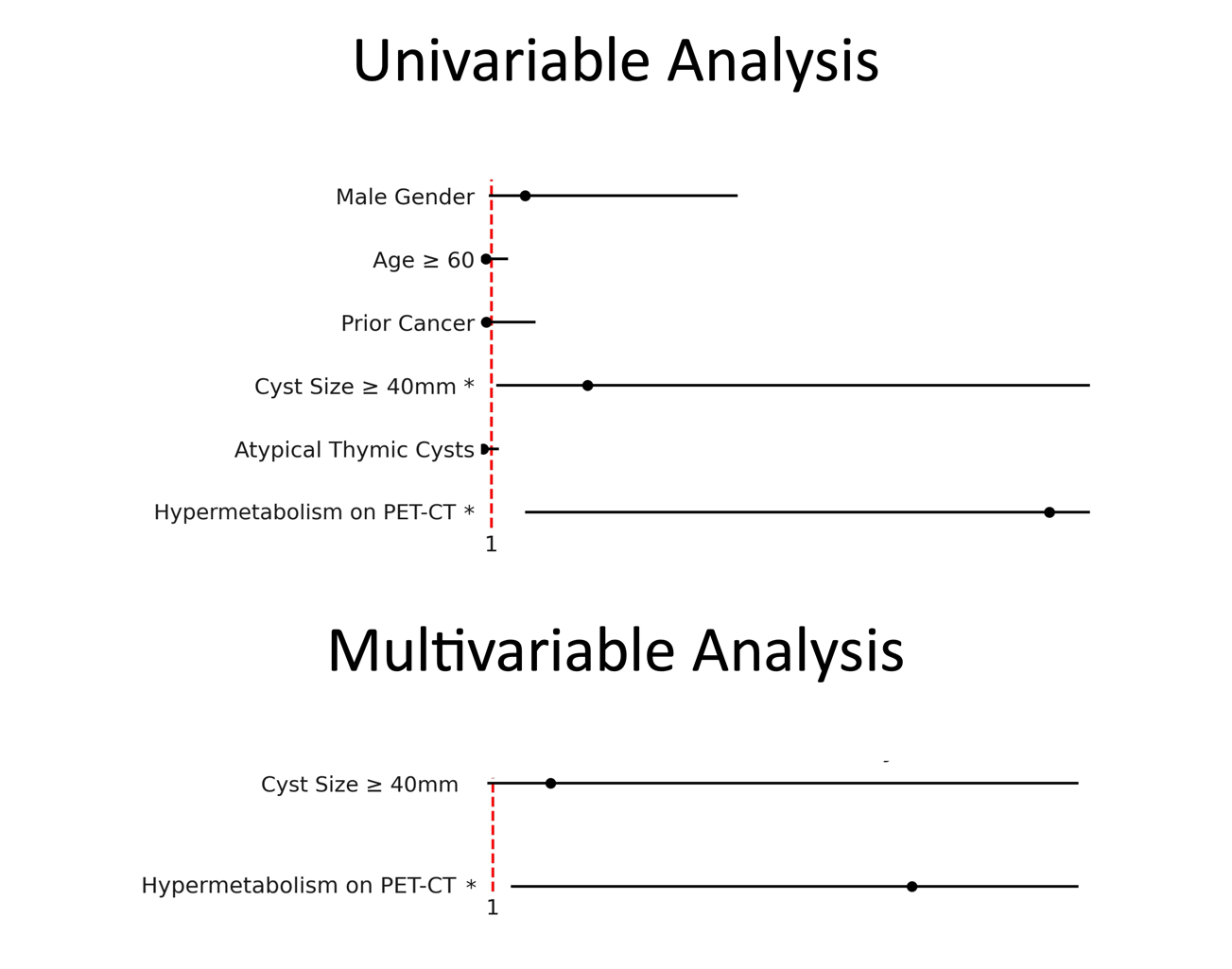


Supplemental Figure 1 : Forest-Plot of univariable and multivariable analyses of predictive factors for malignant lesions or lesions with lesions with malignant potential (i.e. thymoma and germ cell tumors) in pAMC
